# Protein Tyrosine Phosphatase Activity in Insulin-Resistant Rodent *Psammomys Obesus*

**DOI:** 10.1080/15604280214273

**Published:** 2002

**Authors:** Joseph Meyerovitch, Yigal Balta, Ehud Ziv, Joseph Sack, Eleazar Shafrir

**Affiliations:** 1 Pediatric Division Chaim Sheba Medical Center Tel-Hashomer Israel; 2 Sackler School of Medicine Tel Aviv University Tel Aviv Israel; 3 Diabetes Research Unit and Department of Biochemistry Hadassah-Hebrew University Medical Center Jerusalem 91120 Israel

## Abstract

Phosphotyrosine phosphatase (PTPase) activity and its regulation by overnight food deprivation were studied in *Psammomys obesus* (sand rat), a gerbil model of insulin resistance and nutritionally induced diabetes mellitus. PTPase activity was measured using a phosphopeptide substrate containing a sequence identical to that of the major site of insulin receptor (IR) β-subunit autophosphorylation. The PTPase activity in membrane fractions was 3.5-, 8.3-, and 5.9-fold lower in liver, fat, and skeletal muscle, respectively, compared with corresponding tissues of albino rat.Western blotting of tissue membrane fractions in *Psammomys* showed lower PTPase and IR than in albino rats. The density of PTPase transmembrane protein band was 5.5-fold lower in liver and 12-fold lower in adipose tissue. Leukocyte antigen receptor (LAR) and IR were determined by specific immunoblotting and protein bands densitometry and were also found to be 6.3-fold lower in the liver and 22-fold lower in the adipose tissue in the hepatic membrane fractions. Liver cytosolic PTPase activity after an overnight food deprivation in the nondiabetic *Psammomys*  rose 3.7-fold compared with postprandial PTPase activity, but it did not change significantly in diabetic fasted animals. Similar fasting-related changes were detected in the activity of PTPase derived from membrane fraction. In conclusion, the above data demonstrate that despite the insulin resistance, *Psammomys*  is characterized by low level of PTPase activities in membrane and cytosolic fractions in all 3 major insulin responsive tissues, as well as in liver. PTPase activity does not rise in activity as a result of insulin resistance and nutritionally induced diabetes.


*Int. Jnl. Experimental Diab. Res.*, 3:199–204, 2002
Copyright © 2002 Taylor & Francis
1560-4284/02 $12.00 + .00
DOI: 10.1080/15604280290013937

Taylor & Francis healthsciences

### Protein Tyrosine Phosphatase Activity in Insulin-Resistant Rodent *Psammomys obesus*

**Joseph Meyerovitch,[1,2] Yigal Balta,[1,2] Ehud Ziv,[3] Joseph Sack,[1,2] and Eleazar Shafrir[3]**

[1]*Pediatric Division, Chaim Sheba Medical Center, Tel-Hashomer, Israel*
[2]*Sackler School of Medicine, Tel Aviv University, Tel Aviv, Israel*
[3]*Diabetes Research Unit and Department of Biochemistry, Hadassah-Hebrew University Medical Center, Jerusalem, Israel*

**Phosphotyrosine phosphatase (PTPase) activity and its regulation by overnight food deprivation were studied in *Psammomys obesus* (sand rat), a gerbil model of insulin resistance and nutritionally induced diabetes mellitus. PTPase activity was measured using a phosphopeptide substrate containing a sequence identical to that of the major site of insulin receptor (IR) β-subunit autophosphorylation. The PTPase activity in membrane fractions was 3.5-, 8.3-, and 5.9-fold lower in liver, fat, and skeletal muscle, respectively, compared with corresponding tissues of albino rat. Western blotting of tissue membrane fractions in *Psammomys* showed lower PTPase and IR than in albino rats. The density of PTPase transmembrane protein band was 5.5-fold lower in liver and 12-fold lower in adipose tissue. Leukocyte antigen receptor (LAR) and IR were determined by specific immunoblotting and protein bands densitometry and were also found to be 6.3-fold lower in the liver and 22-fold lower in the adipose tissue in the hepatic membrane fractions. Liver cytosolic PTPase activity after an overnight food deprivation in the nondiabetic *Psammomys* rose 3.7-fold compared with postprandial PTPase activity, but it did not change significantly in diabetic fasted animals. Similar fasting-related changes were detected in the activity of PTPase derived from membrane fraction. In conclusion, the above data demonstrate that despite the insulin resistance, *Psammomys* is characterized by low level of PTPase activities in membrane and cytosolic fractions in all 3 major insulin responsive tissues, as well as in liver. PTPase activity does not rise in activity as a result of insulin resistance and nutritionally induced diabetes.**

**Keywords** Insulin Resistance; Nutritionally Iduced Diabetes; Protein Tyrosine Phosphatases; *Psammomys obesus*

Insulin resistance is an innate characteristic of the gerbil *Psammomys obesus*, also known as sand rat [1–3]. Muscle insulin resistance in this desert-adapted animal saves the available food-derived glucose for use by glucose-dependent tissues. The resistance is caused by impaired transduction of the signal through the insulin receptor (IR) to the effector molecules in the cell, associated with overexpression of protein kinase C (PKC) isoenzymes in the skeletal muscle, particularly of PKC$\varepsilon$ [3, 4].

The IR tyrosine kinase (TK), although being activated by insulin, autophosphorylates the IR as well as a number of intracellular proteins that further propagate the insulin signal. Phosphorylation of tyrosine residues on the IR β-subunit in the regulatory domain is critical for maintenance of its activity and, thus, for proper transduction of the signal [5–7]. It has been previously demonstrated that in *Psammomys* placed on high-energy diet, the activation of TK by insulin is impaired [4]. In addition, the overexpressed PKC isoenzymes exert a negative feedback on the transduction of the insulin signal by phosphorylation of serine/threonine residues on IR and other effectors of the insulin signaling pathway [8, 9].

One of the factors affecting the signal transduction is the activity of protein tyrosine phosphatases (PTPases),

Received 29 February 2002; accepted 12 May 2002.
This study was supported by grant no. 2588 from the Chief Scientist's Office of the Ministry of Health, Israel. The authors wish to thank Barry Goldstein, Jefferson Medical College, Pennsylvania, USA, for his interest and advice in the course of this work and Dr. Rony Kalman, Animal Facility of Hebrew University Hadassah Medical School, for the supply of experimental animals.
Address correspondence to Eleazar Shafrir, Department of Biochemistry and Diabetes Research Unit, Hadassah University Hospital, Jerusalem 91120, Israel. E-mail: shafrir@md.huji.ac.il dephosphorylating proteins residing in both cytosolic and membrane cell compartments [10, 11]. The down-regulating role of PTPases in insulin-receptor cascade was demonstrated by findings of increased PTPase activity in tissues of animal models of insulin-resistant obesity and type 2 diabetes, such as obese *fa*/*fa* and diabetic ZDF/Drt-*fa*/*fa* rats [12, 13], and in human obese subjects [14]. The obese humans demonstrated an increase in PTPase leukocyte antigen receptor (LAR) level in subcutaneous fat tissue where this enzyme was found responsible for the elevated total PTPase activity in the membrane fraction [14]. Further, insulin potentiation effect was evident by inhibiting PTPases by vanadate or PTPase-neutralizing antibodies [15–19].

The Middle Eastern desert gerbil becomes diabetic within a few weeks, when being fed a regular laboratory chow that represents a high-energy diet, compared with its natural low-calorie and fiber-rich salt-rich nourishment. The nutritionally evoked diabetes develops through a number of stages. Nondiabetic (fed a low-energy diet) *Psammomys* is considered as stage A. Once placed on high-energy nutrition, the rodent develops hyperinsulinemia (stage B), followed by marked nonfasting hyperglycemia (stage C) [2, 20]. Appearance of high postprandial blood glucose levels is the hallmark for stage C in the course of the disease. Such anomalous predisposition to diet-induced diabetes mellitus is characteristic for most of the *Psammomys* population, with the exception of a selectively inbred diabetes-resistant line [21]. We wished, therefore, to investigate whether an increase in PTPase activity in *Psammomys* might contribute both to the innate insulin resistance and that induced by HE diet.

### MATERIALS AND METHODS

### Materials

The IR $\beta$-subunit–related peptide, TRDIYETDYYRK, was synthesized and purified in the Department of Organic Chemistry of the Weizmann Institute for Science (Rehovot, Israel). Rabbit polyclonal antibodies raised to human LAR and to human IR $\beta$-subunit were supplied by the Transduction Laboratories (Lexington, KY). Goat anti-rabbit immunoglobulin G (IgG)–peroxidase conjugate, protease inhibitors (aprotinin, benzamidine, leupeptin, phenylmethylsulfonyl fluoride [PMSF]), HEPES, Tris, EDTA, NaCl, bovine serum albumin (BSA), ATP, 2-mercaptoethanol, detergents (Tween-20, Triton X-100), and ion-exchanger Dowex $1 \times 8$–400 all were purchased from Sigma (St. Louis, MO). $^{32}$P-labeled ATP was from DuPont (Boston, MA). Acrylamide, *N*,*N*′-methylene-*bis*-acrylamide, sodium dodecyl sulfate (SDS), ammonium persulfate, and TEMED were from BioRad Laboratories (Richmond, CA). The nitrocellulose membranes were from Schleicher & Schuel (Dassel, Germany). Kits for enhanced chemoluminescence procedure (ECL, Amersham, Little Chalfont, Buckinghamshire, England) and for Bradford's protein assay (BioRad), were used.

### Animals

Seven to 8-week-old *Psammomys obesus*, weighing approximately 100 g, from the Hebrew University colony (Jerusalem, Israel) were used. They were held in separate cages and fed ad libitum with special low-calorie nondiabetogenic natural-like diet of 2.3 cal/g [2] (Koffolk, Petach-Tikva, Israel). Wistar albino rats (Harlan Labs) of the same age, weight, and gender, were used as insulin-sensitive reference animals and fed a standard rodent chow (Weizmann Institute). All rodents were normoglycemic, as verified by checking blood glucose levels twice weekly. To induce the stage C hyperinsulinemia-hyperglycemia, 4-week-old male *Psammomys* were placed on the diabetogenic high-energy diet of 3 cal/g for 3 to 4 weeks until at least 2 consecutive non-fasting glucose measurements revealed values of $>300$ mg/dL. Animals, fasted overnight, were sacrificed by cervical dislocation, between 8 and 9 AM. Specimens of liver, leg muscle, and epididymal fat were excised, immediately frozen in liquid nitrogen, and stored at $-80$°C until processed. All experimental procedures were authorized by the Institutional Animal Care Committee.

### Fractionation of Tissues

The preparation of membrane and cytosolic fractions of liver, muscle, and fat was performed as described previously [13, 16]. Briefly, the tissues were homogenized, tissue particles and unbroken cells were removed by low-speed centrifugation (15 minutes at $600 \times g$). The obtained supernatants were ultracentrifuged at $180{,}000 \times g$ for 1 hour and defined as the cytosolic fraction. The resultant pellet was collected, resuspended with a detergent (1% Triton X-100) for 1 hour, and ultracentrifuged ($180{,}000 \times g$, 1 hour). The final supernatant, containing the solubilized membrane proteins, was defined as membrane, or particulate fraction. All procedures were performed at $+4$°C in the presence of protease inhibitors.

### Measurement of PTPase Activity

The PTPase assay was performed as described earlier [13, 22], using the IR-related synthetic phosphopeptide TRDIYETDYYRK, corresponding to amino acids 1142 to 1153 of IR $\beta$-subunit, as substrate. The phosphate released from the peptide was separated from the other chemical forms of radioactive phosphorus by means of organic extraction [23] and quantified with the aid of $\beta$-radioactivity counter. The unit of PTPase activity was defined as picomoles phosphate released per minute per milligram of tissue protein. The determination of total protein concentration in membrane fractions was performed by the Bradford's assay using BSA as a standard [24].

### Immunoblotting

The specimens of membrane fractions containing equal amounts of total protein were run on 6% SDS-polyacrylamide gel electrophoresis (SDS-PAGE) under reducing conditions and transferred onto nitrocellulose sheets. Unbound sites were blocked using 1% BSA in 50 mM Tris, 150 mM NaCl, 0.05% Tween-20, pH 7.4. Rabbit polyclonal anti-LAR or anti–IR $\beta$-subunit antibodies, diluted 1:125 in the blocking solution, were used as primary antibodies. Anti-rabbit IgG-peroxidase conjugate, diluted 1:10,000, was the secondary antibody. Incubation with the antibodies was followed by intensive washing with saline containing 0.05% Tween-20. Protein bands were visualized using ECL detection system. The intensity of the bands was measured using optic densitometer Cliniscan 2 (Helena, Beaumont, TX).

### Statistical Analysis

Data are presented as means ± SE. Groups were compared by paired Student's *t* test.

### RESULTS

The PTPase activity in the membrane and cytosolic preparations is shown in Figure 1. In all 3 major insulin-sensitive tissues, the PTPase activity in both fractions was significantly lower in *Psammomys* compared with albino rats. In liver, the membrane PTPase activity was 1.07 ± 0.38 (n = 8) and 3.76 ± 0.85 (n = 9, $P < .001$) units in *Psammomys* and in albino rats, respectively. In skeletal muscle membrane fraction, the activity was 0.17 ± 0.02 (n = 9) and 1.02 ± 0.27 (n = 7, $P < .001$) units in *Psammomys* and in albino rats, respectively. In membrane preparations from adipose tissue, the enzyme activity was 0.35 ± 0.11 and 2.92 ± 0.91 units in *Psammomys* and in albino rats, respectively (n = 8, $P < .001$), whereas with cytosolic fraction the enzyme activity was 0.19 ± 0.032 (n = 8) and 1.13 ± 0.12 (n = 9, $P < .001$) units in *Psammomys* and in albino rats, respectively. In liver, the cytosolic PTPase activity was 0.31 ± 0.1 (n = 8) and 1.51 ± 0.5 (n = 9, $P < .001$) units in *Psammomys* and in albino rats, respectively. In skeletal muscle cytosolic preparations, the activity was 0.08 ± 0.027 (n = 9) and 0.36 ± 0.046 (n = 7, $P < .001$) units in *Psammomys* and in albino rats, respectively. In both animal species, the highest PTPase activity was found in liver, and the lowest in muscle.

Figure 2 (*upper panels*) shows the immunoblots of liver and fat tissue membrane fractions stained with specific antibodies to the LAR transmembrane subunit. The immunoreactive bands corresponding to the 85-kDa subunit were revealed in the tissues of both rodent species. Optic densitometry showed a significantly lower strength of LAR bands, 5.5-fold lower in liver, and 12-fold lower in fat.

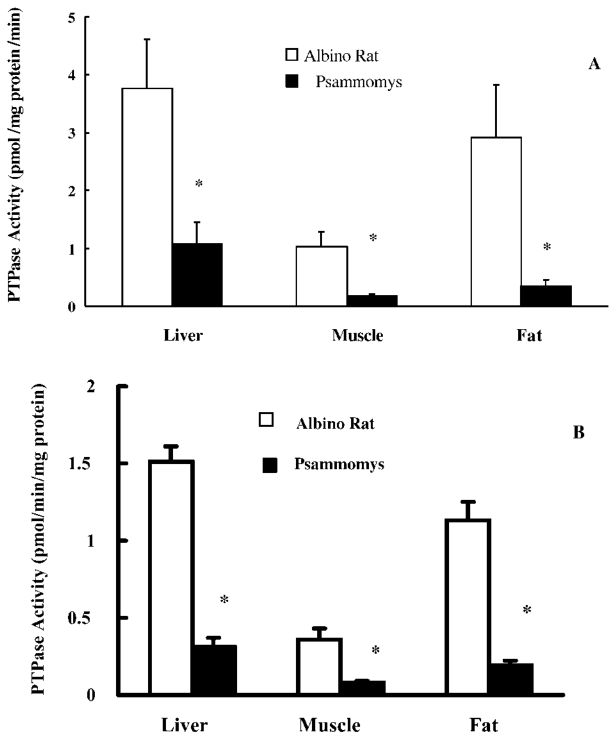

**FIGURE 1**

PTPase activity in insulin responsive tissues of *Psammomys obesus* and albino rats. (*A*) Cytosolic PTPase activity. (*B*) Particulate fractions. Tissues of fasted overnight animals were fractionated using isotonic sucrose differential centrifugation. The particulate fraction was solubilized in Triton X-100 1%. PTPase activity was assayed in cytosolic and in particulate fractions. The PTPase assay was performed using the IR-related synthetic phosphopeptide, corresponding to amino acids 1142 to 1153 of IR $\beta$-subunit, as a substrate. The phosphate released from the peptide was separated from the other chemical forms of radioactive phosphorus by means of organic extraction [22] and quantified with the aid of $\beta$-radioactivity counter. The results represent mean ± SEM of 7 to 9 animals in each group assayed in duplicate in 3 separate experiments. $^{*}P < .01$.

PTPase LAR has been demonstrated to have a high activity towards the IR $\beta$-subunit. For this reason, we assessed the relative amount of the $\beta$-subunits of the IR using immunostaining. The membrane fraction preparations were run on SDS-PAGE, blotted, and immunostained with anti–IR $\beta$-subunit antibodies. The Western blot demonstrated the immunoreactive 97-kDa $\beta$-subunit polypeptides (Figure 2, *lower panels*) in samples from both *Psammomys* and albino rat. The intensity of the IR $\beta$-subunit bands were significantly (6.3 fold) lower in *Psammomys* liver than in the liver of albino rats. In fat, the intensity of these bands was 22 fold lower, thus, the PTPase per IR $\beta$-subunit ratio increased especially in the fat tissue.

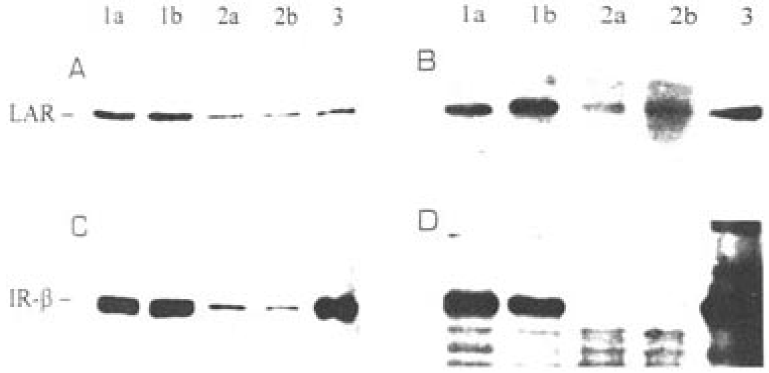

**FIGURE 2**
PTPase LAR immunoblotting and insulin receptor $\beta$-subunit in tissues of Wistar rat and *Psammomys obesus*. Membrane fractions of hepatic and adipose tissues from either species were resolved on SDS/PAGE, transferred onto nitrocellulose paper, and blotted with anti-LAR antibody (panels *A* and *B*) and with anti–IR $\beta$-subunit antibody (panels *C* and *D*). Panels *A* and *C*, liver tissue (50 $\mu$g/lane); panels *B* and *D*, fat tissue (100 $\mu$g/lane). Lanes 1, Wistar rat; lanes 2, *Psammomys obesus*; lanes 3, positive controls for each antibody; letters *a* and *b* refer to duplicates.

LAR PTPase and IR levels in hepatic membrane fractions obtained from nondiabetic *Psammomys obesus* and the diabetes-prone subline were similar to those obtained from diabetic *Psammomys* and from the inbred diabetes-resistant *Psammomys* (data not shown).

An important feature of the nutritionally induced diabetes in *Psammomys obesus* is the low hepatic glucose production during food deprivation. Thus, we studied the regulation of PTPase activity after fasting. The hepatic cytosolic PTPase activity in the normoglycemic (stage A) *Psammomys* was $0.09 \pm 0.025$ units in the fed subgroup, but $0.33 \pm 0.09$ units in the food-deprived subgroup, that is, a 3.7-fold ($n = 8$, $P < .04$) increase was observed. Contrary to this, in the diabetic (stage C) group, the changes after overnight fasting were in the same direction but did not reach statistical significance ($0.15 \pm 0.09$ and $0.26 \pm 0.08$ units, respectively, Figure 3).

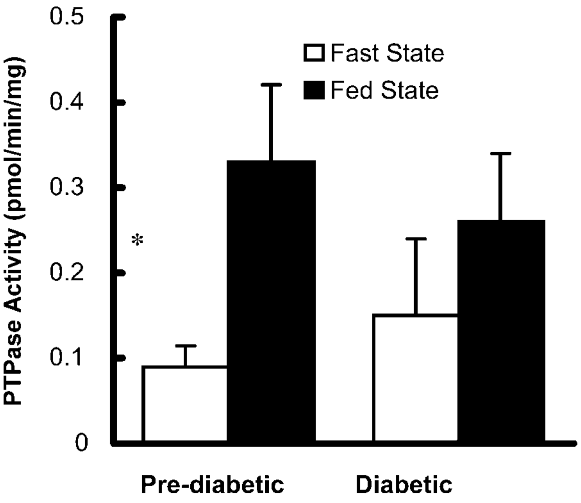

**FIGURE 3**
Effect of overnight food deprivation on PTPase activity of cytosolic fraction of the hepatic tissue in prediabetic and diabetic *Psammomys obesus*. Liver fractionation and PTPase assay as described in Materials and Methods. The results represent mean ± SEM of 9 animals in each group assayed in duplicate. $^*P < .05$.

### DISCUSSION

*Psammomys obesus*, a rodent model of insulin resistance and nutritionally induced diabetes, has been found to have low PTPase activity in both cytosolic and particulate fractions of liver, muscle, and fat, the 3 major insulin-responsive tissues. *Psammomys* also exhibits a low level of the receptor-type PTPase LAR in hepatic and adipose tissues. Low PTPase activity (in comparison with albino rat tissues) should be considered a species characteristics of *Psammomys obesus* in parallel with the low TK activity in muscle and liver of this gerbil, as reported previously [4].

Previous studies regarding PTPase activities, using various substrates and different insulin-resistant states in several genetically diabetic animals, yielded divergent results. In the obese *fa*/*fa* and diabetic ZDF/Drt-*fa*/*fa* rats, an increased in activity of PTPases LAR and PTP 1B, as well as of the SH domain–containing PTPase (SH-PTP2), were found [25, 26]. In mice with type 2–like diabetes, a reduced PTPase activity in both cytosolic and particulate fractions was found using the same enzymatic assay [16]. In human subjects, the basal particulate PTPase activity was 33% higher in insulin-resistant subjects than in insulin-sensitive subjects [25], whereas Kusari and colleagues reported reduced muscle PTPase activity in obese nondiabetic and type 2 diabetic subjects [26].

The lack of contribution of PTPase activity to insulin resistance of *Psammomys* could be attributed to several reasons: A primary defect in the translation or stability of the protein was not proved in this model. The phenotypic characteristic of the homozygous PTPase (−/−) mice with increased insulin sensitivity, and lower plasma levels of insulin and glucose, also do not support a primary defect in the expression of PTPase. The down-regulating of PTPase and cytosolic PTPase secondary to down-regulation of the IR in hyperinsulinemic state is another possible mechanism. However, because there is a reduced level of the IR in the *Psammomys* [4], the ratio of PTPase to the IR does not decrease, or is even increased (in the fat tissue). Thus, the actual steady state of the tyrosine phosphorylation and dephosphorylation may be reduced. The effect of treatment on the *Psammomys* by vanadyl sulphate [27], resulting in reduced blood glucose levels, may indicate that a relative inappropriate overactivity of PTPase is operative in the *Psammomys*.

In our study, we demonstrated the low IR level in *Psammomys obesus* using an immunoblot technique. The low number of IR was also shown by Kanety and colleagues [4] by insulin binding studies. We presume that the low level of IR may render an animal prone to insulin resistance, or it may be secondary to the chronic hyperinsulinemic state characteristic of this phenotype, when maintained on a high-energy diet [1, 2].

With regard to PTP 1B, it was recently found that the expression of this enzyme was elevated in diabetic *Psammomys*; however, PTP 1B specific activity (activity/protein) was significantly decreased in skeletal muscle of diabetic *Psammomys* compared with both the diabetes-resistant line and nondiabetic animals [28]. PTP 1B activity, as well as total PTPase activity, was inversely correlated to serum glucose level, which suggests that PTP 1B, though overexpressed, was attenuated by hyperglycemia or other factors in the diabetic milieu.

Starvation was shown to reduce the activity of liver cytosolic PTPase. Acute regulation of PTPases has also been demonstrated in a hepatoma cell line [29]. However, the diabetic state was associated with poor response of the cytosolic PTPase to fasting, indicating that impaired insulin action is accompanied by change in activity of the enzyme, possibly by a regulatory defect.

The physiologic role of hepatic PTPase may be important in association with hepatic gluconegenesis. Ziv and colleagues [1] found that insulin failed to suppress the hepatic gluconeogenesis in nondiabetic *Psammomys* administered with external insulin. We have demonstrated that vanadate administration suppresses liver gluconeogenesis in the diabetic state of NOD mice, and PTPase may have a role in this metabolic pathway [30].

In conclusion, our data demonstrate that the insulin resistance of *Psammomys*, a desert gerbil surviving on scant nutrition, is characterized by low level of PTPase activity in the membrane and cytosolic fractions in all 3 major insulin-responsive tissues, and an increased response to overnight food deprivation. However, the effect of fasting is lacking in diabetic, insulin-resistant animals. This observation suggests that total PTPase activity does not attenuate IR phosphorylation and its suppression and may serve as an important target for treating the nutritionally induced insulin-resistant state.

### REFERENCES

[1] Ziv, E., Kalman, R., Hershkop, K., Barash, V., Shafrir, E., and Bar-On, H. (1996) Resistance in the NIDDM model *Psammomys obesus* in the normoglycemic, normoinsulinaemic state. *Diabetologia,* **39,** 1269–1275.

[2] Shafrir, E., and Ziv, E. (1998) Cellular mechanism of nutritionally induced insuin resistance: The desert rodent *Psammomys obesus* and other animals in which insulin resistance leads to detrimental outcome. *J. Basic Clin. Physiol. Pharmacol.,* **9,** 347–385.

[3] Shafrir, E., Ziv, E., and Mosthaf, L. (1999) Nutritionally induced insulin resistance and receptor defect leading to $\beta$-cell failure in animal models. *Metabolic Syndrome X,* **892,** 223–246.

[4] Kanety, H., Moshe, S., Shafrir, E., Lunenfeld, B., and Karasik, A. (1994) Hyperinsulinemia induces a reversible impairment in insulin receptor function leading to diabetes in the sand rat model of non-insulin-dependent diabetes mellitus. *Proc. Natl. Acad. Sci. U. S. A.,* **91,** 1853–1857.

[5] Virkamaki, A., Ueki, K., and Kahn, C. R. (1999) Protein-protein interaction in insulin signaling and the molecular mechanisms of insulin resistance. *J. Clin. Invest.,* **103,** 931–943.

[6] Kahn, B. B. (1998) Type 2 diabetes: When insulin secretion fails to compensate for insulin resistance. *Cell,* **92,** 593–596.

[7] White, M. F., and Kahn, C. R. (1994) The insulin signaling system. *J. Biol. Chem.,* **269**(1), 1–4.

[8] Ikeda, Y., Olsen, G. S., Ziv, E., Hansen, L. L., Busch, A. K., Hansen, B. F., Shafrir, E., and Mosthaf-Seedorf, L. (2001) Cellular mechanism of nutritionally induced insulin resistance in *Psammomys obesus*. Overexpression of protein kinase C$\varepsilon$ in skeletal muscle precedes the onset of hyperinsulinemia and hyperglycemia. *Diabetes,* **50,** 584–592.

[9] Bossenmaier, A., Mosthaf, L., Mischak, H., Ullrich, A., and Haring, H. U. (1997) Protein kinase C isoforms $\beta_1$ and $\beta_2$ inhibit tyrosine kinase activity of the insulin receptor. *Diabetologia,* **40,** 863–866.

[10] Sredy, J., Savicki, D. R., Flam, B. R., and Sullivan, D. (1995) Insulin resistance is associated with abnormal dephosphorylation of synthetic phosphopeptide corresponding to the major autophosphorylation sites of the insulin receptor. *Metabolism,* **44,** 1074–1081.

[11] Hashimoto, N., Feener, E. P., Zhang, W. P., and Goldstein, B. J. (1992) Insulin receptor protein-tyrosine phosphatases. Leukocyte common antigen-related phosphatase rapidly deactivates the insulin receptor kinase by preferential dephosphorylation of the receptor regulatory domain. *J. Biol. Chem.,* **2267,** 13811–13814.

[12] Ahmad, F., and Goldstein, B. J. (1995) Increased abundance of specific skeletal muscle protein-tyrosine phosphatases in a genetic model of insulin-resistant obesity and diabetes mellitus. *Metabolism,* **44,** 1175–1184.

[13] Meyerovitch, J., Backer, J. M., and Kahn, C. R. (1989) Hepatic phosphotyrosine phosphatase activity and its alteration in diabetic rats. *J. Clin. Invest.,* **84,** 976–983.

[14] Ahmad, F., Considine, R. V., and Goldstein, B. J. (1995) Increased abundance of the receptor-type protein-tyrosine phosphatase LAR account for the elevated insulin receptor dephosphorylating activity in adipose tissue of obese human subjects. *J. Clin. Invest.,* **95,** 2806–2812.

[15] Meyerovitch, J., Farfel, Z., Sack, J., and Schechter, Y. (1987) Oral administration of vanadate normalizes blood glucose levels in streptozotocin-treated rats. *J. Biol. Chem.,* **262,** 6658–6662.

[16] Meyerovich, J., Rothenberg, P., Shechter, Y., Bonner-Weir, S., and Kahn, C. R. (1991) Vanadate normalizes hyperglycemia in two mouse models of non-insulin-dependent diabetes mellitus. *J. Clin. Invest.,* **87,** 1286–1294.

[17] Ferber, S., Meyerovitch, J., Kriauciunas, K. M., and Kahn, C. R. (1994) Vanadate normalizes hyperglycemia and phosphoenolpyruvate-carboxykinase mRNA levels in ob/obmice. *Metabolism,* **43,** 1346–1354.

[18] Ahmad, F., Li, P.-M., Meyerovitch, J., et al. (1995) Osmotic loading of neutralizing antibodies demonstrates a role for protein-tyrosine-phosphatase 1B in negative regulation of the insulin receptor action pathway. *J. Biol. Chem.,* **270,** 20503–20508.

[19] Kulas, D. T., Zhang, W. R., Goldstein, B. J., Furlanetto, R. W., and Mooney, R. A. (1995) Insulin receptor signaling is augmented by antisense inhibition of the protein tyrosine phosphatase LAR. *J. Biol. Chem.,* **270,** 2435–2438.

[20] Kalderon, B., Gutman, A., Levy, E., Shafrir, E., and Adler, J. H. (1986) Characterization of stages in development of obesity-diabetes syndrome in sand rat (*Psammomys obesus*). *Diabetes,* **35,** 717–724.

[21] Kalman, R., Adler, J. N., Lasarovici, G., et al. (1993) The efficiency of sand rat metabolism is responsible for development of obesity and diabetes. *J. Basic Clin. Physiol. Pharmacol.,* **4,** 57–68.

[22] King, M. G., and Sale, G. J. (1988) Assay of phosphotyrosyl protein phosphatase using synthetic peptide 1142–1153 of the insulin receptor. *FEBS Lett.,* **237,** 137–140.

[23] Shachter, E. (1984) Organic extraction of Pi with isobutanol/toluene. *Anal. Biochem.,* **138,** 416–420.

[24] Bradford, M. (1976) A rapid and sensitive method for the quantitation of microgram quantities of protein utilizing the principle of protein dye binding. *Anal. Biochem.,* **72,** 248–254.

[25] McGuire, M. C., Fields, R. M., Nyomba, B. L., Raz, I., Bogardus, C., Tonks, N. K., and Sommercorn, J. (1991) Abnormal regulation of protein tyrosine phosphatase activities in skeletal muscle of insulin-resistant humans. *Diabetes,* **40,** 939–942.

[26] Kusari, J., Kenner, K. A., Suh, K. I., Hill, D. E., and Henry, R. R. (1994) Skeletal muscle protein tyrosine phosphatase activity and tyrosine phosphatase 1B protein content are associated with insulin action and resistance. *J. Clin. Invest.,* **93,** 1156–1162.

[27] Shafrir, E., Spielman, S., Nachliel, I. Khamaisi, M., Bar-On, H., and Ziv, E. (2001) Treatment of diabetes with vanadium salts: General overview and amelioration of nutritionally induced diabetes in the *Psammomys obesus* gerbil. *Diabetes/Metab. Res. Rev.,* **17,** 55–66.

[28] Ikeda, Y., Ziv, E., Shafrir, E., and Mosthaf-Seedorf, L. (2002) Protein tyrosine phosphatase 1B is impaired in skeletal muscle of diabetic *Psammomys obesus*. *Int. J. Exp. Diabetes Res.,* **3,** 205–212.

[29] Meyerovitch, J., Backer, J. M., Csermely, P., Shoelson, S. E., and Kahn, C. R. (1992) Insulin differentially regulates protein phosphotyrosine phosphatase activity in rat hepatoma cells. *Biochemistry,* **31,** 10338–10344.

[30] Mossery, R., Waner, T., Shefi, M., Shafrir, J. E., and Meyerovitch, J. (2000) In vivo effects of vanadate treatment on hepatic glucose-6-phosphatase in non obese diabetic (NOD) mice model. *Metabolism,* **49,** 321–325.

